# Long-Term Visual Outcomes of Secondary Intraocular Lens Implantation in Children with Congenital Cataracts

**DOI:** 10.1371/journal.pone.0134864

**Published:** 2015-07-31

**Authors:** Xianfang Rong, Yinghong Ji, Yanwen Fang, Yongxiang Jiang, Yi Lu

**Affiliations:** 1 Department of Ophthalmology and Vision Science, Eye and ENT Hospital of Fudan University, Shanghai, China; 2 Key Laboratory of Myopia of State Health Ministry, and Key Laboratory of Visual Impairment and Restoration of Shanghai, Shanghai, China; Sun Yat-sen University, CHINA

## Abstract

**Aim:**

The aim of this study was to evaluate the long-term visual outcomes and factors affecting visual results in children undergoing secondary intraocular lens (IOL) implantation following primary congenital cataract extraction.

**Methods:**

Children with congenital cataracts who underwent secondary IOL implantation for aphakia at the Eye and ENT Hospital of Fudan University between January 1, 2001, and December 31, 2007, were retrospectively reviewed. One eye was randomly selected in children with bilateral cataracts. Laterality, type of cataract (total or partial opacity), sex, age at primary and secondary surgeries, visual axis opacity (VAO), compliance with amblyopia therapy, postoperative ocular complications, refractive error, ocular alignment, and best corrected visual acuity (BCVA) at last follow-up were recorded; potential factors that might have affected the visual results were evaluated.

**Results:**

Seventy-six bilateral and 34 unilateral congenital cataract cases were analyzed; the mean ages of the children were 12.70±5.06 and 12.50±2.71 years at final follow-up, the mean follow-up periods from IOL implantation were 94.93±24.22 and 109.09±18.89 months, and the mean BCVA (Log MAR) values were 0.51±0.37 and 1.05±0.46, respectively. Final BCVA after secondary IOL implantation was significantly associated with laterality, type of cataract, age at primary cataract extraction, compliance with amblyopia therapy, and refractive correction after surgery. No significant associations were found between BCVA and sex, age at secondary IOL implantation, VAO, or other ocular complications. The most common ocular complications were VAO and elevated intraocular pressure after surgery. There were no other complications, with the exception of one eye with IOL dislocation.

**Conclusions:**

The results indicate that the important determinants of long-term visual outcomes in children with congenital cataracts undergoing secondary IOL implantation are laterality, cataract type, age at initial cataract extraction, compliance with amblyopia therapy, and refractive error.

## Introduction

Congenital cataract, characterized by visual deprivation and formation of amblyopia, is an important, treatable cause of childhood visual handicap throughout the world [1]. A better understanding of the sensitive periods in visual development and the timing of cataract removal are critical for the reversal of amblyopia; early intervention is recommended. In children with congenital cataracts, the amblyogenic window for successful visual rehabilitation is probably within the first three months of life [2,3]. In normal children, axial length increases rapidly until 2–3 years of age [4], and intraocular lens (IOL) implantation after cataract extraction is more susceptible to myopic shift in children under the age of 2 years. These children are also more likely to suffer from intensive posterior capsule opacification (PCO) and excessive uveal inflammation [5,6]. Because IOL implantation in these children still remains controversial, primary cataract extraction is preferred in early detection cases, followed by spectacles or contact lenses to correct surgical aphakia, combined with management of amblyopia [7,8]. The Infant Aphakia Treatment Study has shown that there is no difference in visual acuity between children with unilateral cataracts undergoing primary IOL implantation and those prescribed contact lenses to correct surgical aphakia [6]. Secondary IOL implantation is considered when an aphakic child over 2 years of age becomes intolerant to wearing contact lenses or spectacles [9]. However, few studies have focused on the long-term visual outcomes of secondary IOL implantation, and the long-term visual results have not been determined. Thus, the goal of the current study was to investigate the long-term visual results of secondary IOL implantation in aphakic children over 2 years of age, as well as the potential factors affecting postoperative visual acuity.

## Materials and Methods

Children diagnosed with congenital cataracts at the Eye and ENT Hospital of Fudan University, Shanghai, China, who underwent secondary IOL implantation between January 1, 2001, and December 31, 2007, were included in this retrospective study. All of the children had previously undergone manual anterior capsulorhexis, irrigation and aspiration of cataracts, posterior capsulectomy, and anterior vitrectomy. The secondary postponed IOL implantations were performed via a 2.6–3.2mm superior limbal tunnel incision. A three-piece hydrophobic, acrylic, foldable IOL was placed in the ciliary sulcus, and an anterior vitrectomy was performed. The tunnel incision was closed with one 10–0 nylon suture. After surgery, the operated eyes were treated with topical antibiotics, corticosteroids, and non-steroidal anti-inflammatory drugs. All of the surgeries (including initial cataract extraction and secondary IOL implantation) were performed by the same experienced surgeon (Yi Lu), with the patients under general anesthesia. Children with ocular defects such as traumatic cataract, retinopathy of prematurity, persistent fetal vasculature, congenital glaucoma, microphthalmos, Marfan’s syndrome, or other anterior or posterior segment anomalies, were excluded from the study. Children with systemic diseases that could influence learning ability were also excluded. One eye was randomly selected in children with bilateral cataracts.

The children’s cataracts were categorized according to opacity type—total or partial. Cataracts that impeded vision of the red reflex during an ocular fundus examination were considered total. For both the initial and secondary surgeries, the patients were examined preoperatively and postoperatively at one day, one week, two weeks, and one month, and then at six-month intervals until the last follow-up. All aphakic children were prescribed spectacles or contact lenses combined with management of amblyopia. Visual axis opacity (VAO), including PCO, was considered pre-VAO when it developed after the initial cataract extraction and post-VAO when it developed after the IOL implantation surgery. Compliance with amblyopia therapy was reported as none, poor, or good if 0–25%, 25–75%, or 75–100%, respectively, of the prescribed hours were reported.

The patients underwent ophthalmological examinations that included intraocular pressure (IOP) measurement with a Tono-Pen or non-contact tonometer and strabismus examination with a Krimsky test, alternative cover test, or Hirschberg test. Other signs, such as nystagmus, were also reported. Refractive error at last follow-up was calculated as the spherical equivalent refraction, using the algebraic power of the sphere plus half the cylindrical power. Absolute value of refractive error was used for analysis. Best corrected visual acuity (BCVA) was determined using a standard crowded Snellen chart and converted to the logarithm of the minimum angle of resolution (Log MAR) for statistical analysis.

This study was prospectively approved by the Ethics Committee of the Eye and ENT Hospital, Fudan University, Shanghai, China. All patient information was collected after obtaining written informed consent from the children’s guardians on their behalf. The consent procedure was also approved by the ethics committee. All procedures adhered to the tenets of the Declaration of Helsinki for research involving human subjects.

### Statistical analysis

The difference in final BCVA between the bilateral and unilateral children was analyzed with a Mann–Whitney *U* test. Linear regression analysis was used to evaluate the associations between potential factors and BCVA at last follow-up, using Stata statistical software version 9.0 (Stata Corporation, College Station, TX). Initially, factors that could influence visual outcome, such as sex, cataract type, age at initial cataract extraction, age at secondary IOL implantation, compliance with amblyopia therapy, pre-VAO, post-VAO, refractive error at last follow-up, and other complications after IOL implantation were investigated using univariate linear regression analysis. Then, multivariate linear stepwise regression analysis was performed to identify influential factors. Interaction terms were checked with the multivariate analysis model, taking into account all other factors, as there were factors that were not significant in the univariate analysis. Bilateral and unilateral cases were analyzed separately, due to the inherent differences in terms of factors related to visual outcomes and amblyopia. The data were presented as mean ± standard deviation; p<0.05 was considered statistically significant.

## Results

From 2001 to 2007, 110 eyes underwent secondary IOL implantation surgery, including 76 bilateral and 34 unilateral cases. The children’s demographics are listed in Table 1. In patients with bilateral congenital cataracts, mean age at cataract extraction was 7.45±4.73 months, and age at secondary IOL implantation was 46.64±29.37 months. Mean follow-up period was 94.93±24.22 months, and age at last follow-up was 12.70±5.06 years. In unilateral cases, mean age at cataract extraction was 8.32±4.91 months, and age at secondary IOL implantation was 33.82±16.43 months. Mean follow-up period was 109.09±18.89 months, and age at last follow-up was 12.50±2.71 years. Mean refractive error at last follow-up was -3.11±2.45 diopters (D) in bilateral cases and -2.39±2.77D in unilateral cases. Mean BCVA (Log MAR) at last follow-up was significantly better in bilateral cases (0.51±0.37) than in unilateral cases (1.05±0.46); with p<0.001.

**Table 1:** Children’s demographics.

	Laterality	
	Bilateral	Unilateral
Number of children	76	34
Sex		
Male	50	15
Female	26	19
Cataract opacity type		
Total	47	17
Partial	29	17
Age at cataract extraction(months)	7.45±4.73 (range, 3–31)	8.32±4.91 (range, 3–23)
Age at IOL implantation(months)	46.64±29.37 (range, 23–184)	33.82±16.43 (range, 22–106)
Age at last follow-up(years)	12.70±5.06 (range, 8–27)	12.50±2.71 (range, 9–18)
Mean follow-up from IOL implantation (months)	94.93±24.22 (range, 65–166)	109.09±18.89 (range, 68–168)
Mean BCVA (Log MAR)	0.51±0.37	1.05±0.46
Refractive error at last follow-up (D)	-3.11±2.45 (range, -7.50–+2.25)	-2.39±2.77 (range, -7.50–+3.00)

The univariate analysis results showed that in both bilateral and unilateral cataract cases, cataract type, age at cataract extraction, compliance with amblyopia therapy after surgery, and refractive error were significantly associated with BCVA at last follow-up (Tables 2 and 3), but not sex, age at secondary IOL implantation, pre-VAO, post-VAO, or presence of other complications post-secondary IOL implantation. Better visual outcome was achieved in children with partial opacity cataracts extracted at an early age. Good compliance with amblyopia treatment and low post-surgical refractive error also contributed to better visual acuity.

**Table 2:** Univariate analysis of factors associated with visual acuity (BCVA Log MAR) in bilateral cataract cases.

Variables	Standardized β	t	P value
Sex	0.045	0.391	0.697
Cataract type	-0.317	-2.878	0.005
Age at cataract extraction	0.259	2.303	0.024
Age at IOL implantation	-0.062	-0.538	0.592
Compliance with amblyopia therapy	-0.428	-4.729	<0.001
Pre-VAO†	0.156	1.360	0.178
Post-VAO‡	0.082	0.709	0.481
Refractive error at last follow-up	0.267	2.385	0.020
Presence of other complications post IOL implantation^1^	0.085	0.733	0.466

†Visual axis opacity developed prior to secondary IOL implantation and after initial cataract extraction.

‡Visual axis opacity developed after secondary IOL implantation surgery.

^1^ Presence of other complications (elevated IOP and IOL dislocation) post IOL implantation.

**Table 3:** Univariate analysis of factors associated with visual acuity (BCVA Log MAR) in unilateral cataract cases.

Variables	Standardized β	t	P value
Sex	-0.111	-0.632	0.532
Cataract type	-0.627	-4.552	<0.001
Age at cataract extraction	0.732	6.077	<0.001
Age at IOL implantation	-0.141	-0.805	0.427
Compliance with amblyopia therapy	-0.385	-2.362	0.024
Pre-VAO†	-0.232	-1.351	0.186
Post-VAO‡	-0.133	-0.757	0.455
Refractive error at last follow-up	0.738	6.182	<0.001
Presence of other complications post IOL implantation^1^	-0.238	-1.386	0.175

†Visual axis opacity developed prior to secondary IOL implantation and after initial cataract extraction.

‡Visual axis opacity developed after secondary IOL implantation surgery.

^1^ Presence of other complications (elevated IOP and IOL dislocation) post IOL implantation.

Multivariate linear stepwise regression analysis further revealed that neither the bilateral nor the unilateral children’s visual outcomes were significantly associated with sex, age at secondary IOL implantation surgery, pre-VAO, post-VAO, or presence of other ocular complications after IOL implantation. Final BCVA differed significantly between total and partial opacity cataract cases (p = 0.002 and p = 0.005, respectively, for bilateral and unilateral cases). The children who underwent cataract extraction at an early age and those who complied well with amblyopia therapy had much better visual acuity than those who did not. The results also revealed that refractive error post-IOL implantation was an essential factor in visual outcome (Tables 4 and 5).

**Table 4:** Multivariate analysis of factors associated with visual acuity (BCVA Log MAR) in bilateral cataract cases.

Variables	Standardized β	t	P value
Cataract type	-0.295	-3.212	0.002
Age at cataract extraction	0.215	2.359	0.021
Compliance with amblyopia therapy	-0.499	-5.599	<0.001
Refractive error at last follow-up	0.194	2.059	0.043

**Table 5:** Multivariate analysis of factors associated with visual acuity (BCVA Log MAR) in unilateral cataract cases.

Variables	Standardized β	t	P value
Cataract type	-0.310	-3.047	0.005
Age at cataract extraction	0.361	3.015	0.005
Compliance with amblyopia therapy	-0.218	-2.392	0.023
Refractive error at last follow-up	0.309	2.498	0.018

The children’s final BCVA scores (Snellen equivalent) are summarized in Table 6. Median BCVA at last follow-up was 20/50 in children with bilateral cataracts; 7.9% of the children had visual acuity worse than 20/200. In unilateral cases, the median BCVA was 20/200; 47.1% of the children had visual acuity worse than 20/200. The distribution of children with BCVA<20/200 among factors that significantly influenced visual outcome was also analyzed, and the results are shown in Table 7. In the unilateral cataract cases, patients with total opacity cataracts had a much higher percentage of bad visual acuity (76.5%), as did patients who underwent cataract intervention when they were older than 8 months (81.3%), patients with no or poor amblyopia therapy compliance (66.7% and 50.0%, respectively), and patients with high ocular refractive error (87.5%). In these children, even with good compliance with amblyopia therapy, 36.4% of the cases had visual acuity worse than 20/200. These results further confirmed that the factors were essentially associated with vision rehabilitation after cataract surgery.

**Table 6:** Distribution of BCVA (Snellen equivalent) of secondary IOL implantation cases at last follow-up.

BCVA at last follow-up	Number and percentage of eyes	
	Bilateral	Unilateral
20/20≤VA	4(5.3%)	0(0%)
20/25≤VA<20/20	9(11.8%)	0(0%)
20/50≤VA<20/25	26(34.2%)	5(14.7%)
20/100≤VA<20/50	18(23.7%)	4(11.8%)
20/200≤VA<20/100	13(17.1%)	9(26.5%)
VA<20/200	6(7.9%)	16(47.1%)
Total	76(100%)	34(100%)

**Table 7:** Distribution of BCVA<20/200 among the associated factors in secondary IOL implantation cases at last follow-up.

Factors associated with bad BCVA	Number and Percentage (%) of BCVA<20/200	
	Bilateral	Unilateral
Cataract type		
Total	6/47(12.8%)	13/17(76.5%)
Partial	0/29(0%)	3/17(17.6%)
Age at cataract extraction		
≤8 months	3/60(5%)	3/18(16.7%)
>8 months	3/16(18.6%)	13/16(81.3%)
Compliance of amblyopia therapy		
None	2/18(11.1%)	5/6(66.7%)
Poor	4/22(18.2%)	3/6(50.0%)
Good	0/36(0%)	8/22(36.4%)
Refractive error at last follow-up		
≤3.00 D	2/33(6.1%)	2/18(11.1%)
>3.00 D	4/43(9.3%)	14/16(87.5%)

In the overall group of 110 children, no further post-surgical complications occurred during the period between cataract extraction and IOL implantation, with the exception of pre-VAO in 13 children (11.8%). Complications after secondary IOL implantation included post-VAO in six children (5.5%), elevated IOP in six children (5.5%), and IOL dislocation in one child (0.9%). No other complications were observed after secondary IOL implantation. All VAOs were removed by anterior vitrectomy or Nd:YAG laser capsulotomy immediately after being detected; elevated IOP was controlled with drugs, and IOL dislocation was corrected by reoperation.

Among the 110 children, strabismus was present in 39 eyes (35.5%) and nystagmus was present in 69 eyes (62.7%) at last follow-up. In the children with bilateral cataracts, strabismus was present in 31.6% and nystagmus in 60.5% of the eyes. In unilateral cases, the incidence rates of strabismus and nystagmus were 44.1% and 67.6%, respectively.

## Discussion

While several studies on visual results post-congenital cataract surgery have been conducted over the past 20 years, few studies have been conducted on long-term visual outcome in children with secondary IOL implantation after congenital cataract removal. As such, in this study, long-term visual acuity was explored in children with congenital cataracts who underwent secondary IOL implantation after cataract extraction, and the factors associated with visual results were analyzed. Congenital cataract treatments are long-term, complex, and intensive; it has been reported that long-term visual outcome can be predicted at 7 years of age [10]. The mean follow-up periods from IOL implantation in this study were 94.93±24.22 and 109.09±18.89 months in bilateral and unilateral cases, respectively, and the mean ages at final follow-up were 12.70±5.06 years in bilateral cases and 12.50±2.71 years in unilateral cases; the results were appropriate for evaluating long-term visual outcome. The findings also provided the clinicians and parents with data to assist in determining whether the intended therapeutic approaches could lead to improved visual outcome.

Previous studies have demonstrated that myopic shift and ocular complications are likely to occur in children who were younger than 2 years of age when they underwent IOL implantation [5,11,12]. Secondary IOL implantation is an optimal option for aphakic children who underwent cataract removal at an early age; IOLs can be implanted when optical correction with contact lenses or spectacles is resisted or when additional correction is not required. In this study, refractive error values at last follow-up were -3.11±2.45 D (range, -7.50–+2.25 D) in bilateral cataract cases and -2.39±2.77 D (range, -7.50–+3.00 D) in unilateral cases (Table 1), which was consistent with recent studies on secondary IOL implantation by Maqli et al. (2013) and Kim et al. (2012) [12,13]. Maqli et al. also found that myopic shift was more frequent in eyes that underwent primary IOL implantation compared with those that underwent secondary IOL implantation at an older age [12].

To reduce surgery-induced biases and biases caused by different surgeons, only children whose surgeries were performed by the same skilled surgeon (Yi Lu) were included in this study. Children with complicated cataract cases, such traumatic cataract, persistent fetal vasculature, microphthalmos, or other anterior or posterior segment anomalies, were excluded. The data analysis indicated that long-term visual outcomes were associated with laterality, cataract type, age at primary cataract extraction, and compliance with amblyopia therapy (Tables 1–5). Children with bilateral cataracts had better visual acuity than those with unilateral disease (Tables 6 and 7). This might be due to the fact that an infant’s eyes compete for synaptic contacts in the cortex. Specifically, in binocular cataract, both eyes convey similar blurred images to the brain, resulting in equal synaptic connections for both eyes. In monocular cataract, however, the eye with the cataract (later the aphakic or pseudophakic eye) is inferior to the normal phakic eye in image sharpness and it therefore develops fewer synaptic connections in the brain and will be more prone to amblyopia [14]. Partial opacity also contributed to good visual results. Early surgical intervention, good compliance with amblyopia treatment, and optical correction of refractive error were found to be essential for vision rehabilitation (Tables 2–5, 7). The results of this study also revealed that the child’s age at secondary IOL implantation was not associated with final BCVA. This finding might due to the fact that all aphakic children were prescribed correction of refractive error after cataract extraction. However, in both bilateral and unilateral cases, BCVA was significantly associated with refractive error at last follow-up. Parents’ negligence regarding refractive error correction post-IOL implantation might be responsible for the unfavorable result. The results demonstrated the importance of refractive error correction throughout the process of visual rehabilitation in children with congenital cataracts. Medical education for the parents regarding the importance of refractive error correction was indispensable and was still followed long after IOL implantation. These findings correspond with a secular trend of good visual outcomes, which might be due to a combination of improved amblyopia management through earlier surgery, increased emphasis on occlusion, and better correction of postoperative refractive error [15]. The findings are supported by previous study results [16–19].

In this study, aphakia in children after cataract removal was corrected optically. VAOs (including PCO) were removed surgically or by Nd:YAG laser immediately after detection during follow-up, and other ocular complications such as elevated IOP and IOL dislocation were addressed in a timely manner. This prompt treatment might explain why age at secondary IOL implantation surgery, pre-VAO, post-VAO, and other complications after IOL implantation did not influence the final visual outcomes.

It has been reported that 69% of children with primary IOL implantation had VAO formation and 63% needed additional intraocular surgery during the first postoperative year [20]. In addition, glaucoma has been reported in 6–63% of children after congenital cataract extraction [13,21,22]. In the current study, thirteen eyes (11.8%) developed pre-VAO after primary cataract extraction, six eyes (5.5%) developed post-VAO after secondary IOL implantation, and six eyes (5.5%) developed elevated IOP. The Results revealed that the occurrence of post-operation complications in the current study was much lower compared with the previous studies. Thus, it could be concluded that the approaches used to treat congenital cataracts in this study were much safer, possibly due to delayed IOL implantation and improved surgical techniques.

Recent studies regarding postoperative visual outcome in bilateral congenital cataract cases have reported median final BCVA values of 20/60 following primary IOL implantation [23] and 20/50 following secondary IOL implantation [13]. In the current study, the median BCVA in bilateral cases at last follow-up was 20/50, which was consistent with previous studies. However, the final median BCVA following secondary IOL implantation in unilateral cases was 20/200 in the current study, which was almost the same as previous studies on primary IOL implantation in unilateral cases [23]. This result might be due to the fact that children with unilateral cataracts often have worse visual acuity than patients with bilateral disease, as was found in the current study.

Strabismus and nystagmus are well-known consequences in children with congenital cataracts, with reported frequencies of 27%~100% [24–26] and 71% [26], respectively. In the current study, strabismus was present in 35.5% of the included eyes, and nystagmus was present in 62.7%. The frequency rates of strabismus and nystagmus were 31.6% and 60.5%, respectively, in bilateral cataract cases, and 44.1% and 67.6%, respectively, in unilateral cases. It has been reported that the risks of strabismus and nystagmus are higher in children with unilateral cataracts than in children with bilateral cataracts, as well as in children with worse visual outcomes [26,27]. These high rates of strabismus and nystagmus in infantile cataract cases might be due to visual deprivation during the critical period of visual development. The presence of strabismus and nystagmus has been shown to be associated with a worse visual outcome in children with dense congenital cataracts, and it is less likely to develop in children whose cataracts were removed at an earlier age [25–27].

In conclusion, the results of this study revealed that long-term visual outcomes were associated significantly with laterality, cataract type, age at primary cataract extraction, compliance with amblyopia therapy and refractive error post-secondary IOL implantation. Better visual outcomes were achieved in children with bilateral cataracts of partial opacity, close adherence to amblyopia therapy, and optical correction of refractive error, both post-primary cataract extraction and post-secondary IOL implantation surgery. The findings serve to remind clinicians and parents that although cataract laterality and opacity type cannot be changed in children with congenital cataracts, early surgical intervention, good compliance with amblyopia treatment, and optical correction of refractive error count a great deal, and they can be achieved for vision rehabilitation. The data also revealed that secondary IOL implantation resulted in a lower rate of ocular complications and less myopia shift. Limitations of this study were the retrospective design and relatively small sample size, especially in the group with unilateral cataracts. Further investigation might be necessary to compare long-term ocular outcomes, such as visual acuity, refractive error, ocular complications, ocular fixation control, and eye alignment, between primary and secondary IOL implantation groups and between bilateral and unilateral groups. Treating congenital cataracts still remains a challenge.
